# Traditional Chinese Medicine Integrated Responsive Microneedles for Systemic Sclerosis Treatment

**DOI:** 10.34133/research.0141

**Published:** 2023-05-09

**Authors:** Xi Luan, Xiaoxuan Zhang, Min Nie, Yuanjin Zhao

**Affiliations:** ^1^ Department of Rheumatology and Immunology, Nanjing Drum Tower Hospital, School of Pharmacy, Clinical College of Traditional Chinese and Western Medicine, Nanjing University of Chinese Medicine, Nanjing 210023, China.; ^2^ State Key Laboratory of Bioelectronics, School of Biological Science and Medical Engineering, Southeast University, Nanjing 210096, China.

## Abstract

Traditional Chinese medicine, such as *Tripterygium wilfordii* and *Paeonia lactiflora*, has potential values in treating systemic sclerosis (SSc) and other autoimmune diseases, while their toxic side effect elimination and precise tropical drug delivery are still challenges. Here, we present multiple traditional Chinese medicine integrated photoresponsive black phosphorus (BP) microneedles (MNs) with the desired features for the SSc treatment. By employing a template-assisted layer-by-layer curing method, such MNs with triptolide (TP)/paeoniflorin (Pae) needle tips and BP-hydrogel needle bottoms could be well generated. The combined administration of TP and Pae can not only provide anti-inflammatory, detoxification, and immunomodulatory effects to treat skin lesions in the early stage of SSc but also remarkably reduce the toxicity of single drug delivery. Besides, the additive BPs possess good biocompatibility and near-infrared (NIR) responsiveness, imparting the MN photothermal-controlled drug release capability. Based on these features, we have demonstrated that the traditional Chinese medicine integrated responsive MNs could effectively improve skin fibrosis and telangiectasia, reduce collagen deposition, and reduce epidermal thickness in the SSc mouse models. These results indicated that the proposed Chinese medicine integrated responsive MNs had enormous potential in clinical therapy of SSc and other diseases.

## Introduction

Systemic sclerosis (SSc) is a kind of autoimmune disease characterized by small-vessel disease and fibroblast dysfunction, which has poor prognosis and high mortality [1,2]. Early treatment plays an important role in improving the survival rate of SSc, and skin lesion is the main manifestation of SSc in the early stage [3,4]. In clinical practices, medication is the kingcraft for SSc treatment. Many Western medicines, including glucocorticoids, immunosuppressants, and methotrexate, have been frequently used, while their therapeutic effects are usually unsatisfied [5–8]. As an alternative, traditional Chinese medicine, such as *Tripterygium wilfordii* and *Paeonia lactiflora*, has attracted more and more attention from clinicians due to its strong immunosuppressant effects [9–13]. However, these drugs are normally administered orally or intravenously at present, which are difficult to act effectively on the lesions in the skin and lead to different degrees of systemic adverse reactions [5]. Besides, although some dosage forms have been developed for local external application, their clinical use is still limited by simple composition and single function [14]. Thus, it remains a great challenge to reduce the traditional Chinese medicine toxicity and improve its delivery efficiency for SSc treatment [15–19].

**Fig. 1.**
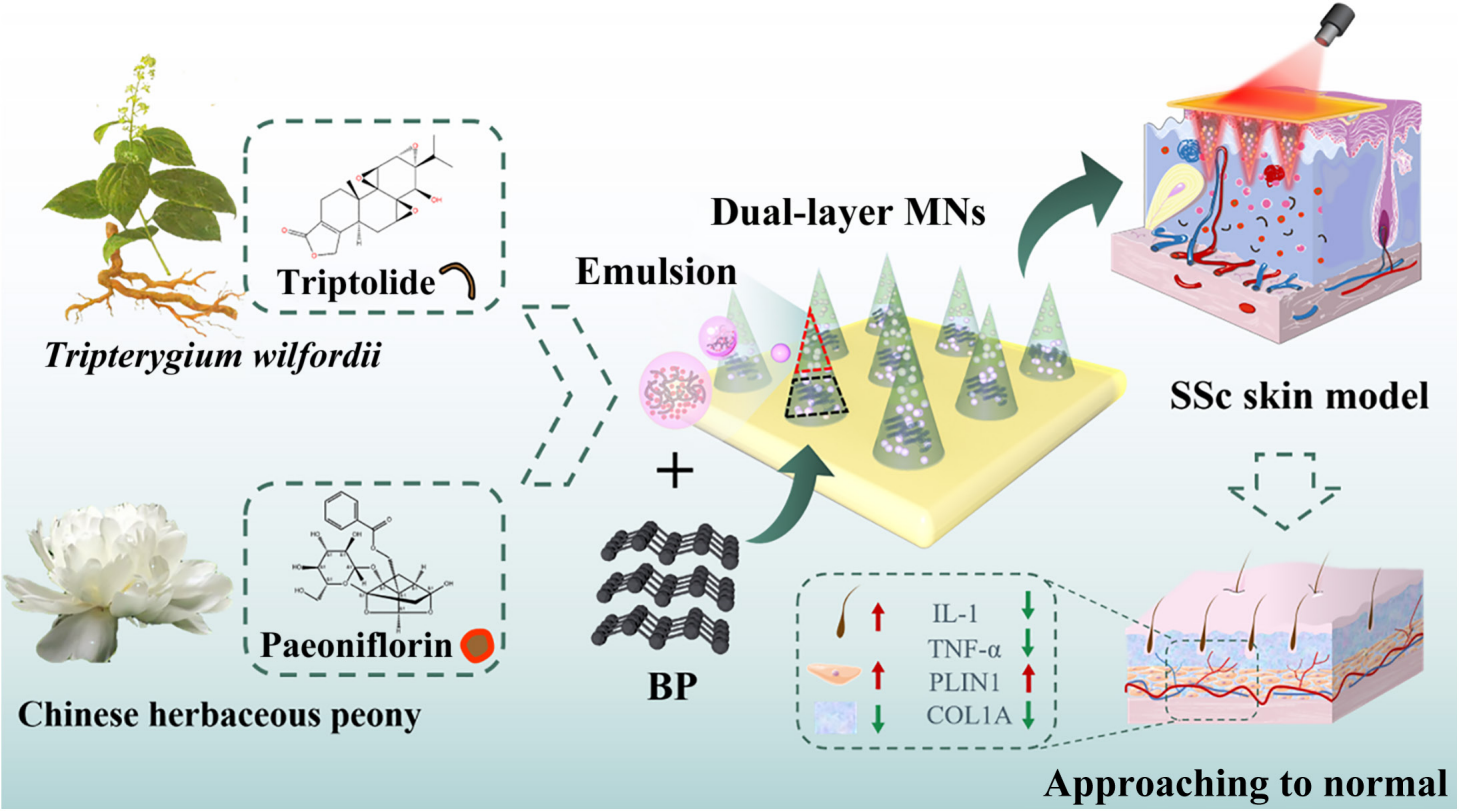
Schematic illustrations of NIR response MNs loaded with TP and Pae for the treatment of early skin lesions of SSc.

Here, we proposed novel photoresponsive black phosphorus (BP) microneedles (MNs) with dual traditional Chinese medicine integration for treating SSc skin injury, as shown in Fig. 1. As an emerging transdermal delivery method for local drug administration, MNs have proven their ability to penetrate the cuticle, act on the deep tissue of lesions directly, avoid liver first-pass effects, enhance drug absorption, and reduce the systemic adverse reactions [20–26]. In contrast, characterized by good biocompatibility, low cytotoxicity, ideal biodegradability, and near-infrared (NIR) responsiveness, BPs enabled the photothermal regulation of their tagged hydrogels or other delivery systems to release the actives quickly and controllably [27–35]. Benefiting from these advantages and properties, the integration of MNs and BPs has been regarded as an intelligent delivery stratagem to skin and becomes a new research direction [34,36–40]. However, these technologies are seldom integrated with traditional Chinese medicine, and the reported examples are usually with single function of delivering one single pharmaceutical, which have restricted their practical values [21,25,41]. In addition, the efforts of these MNs in treating SSc are still lacking investigation.

In this paper, we present the desired photoresponsive multifunctional MNs by integrating different traditional Chinese medicine and BP nanoparticles, and explore their values in topical treatment of SSc. By employing a template-assisted layer-by-layer curing method, such MNs with triptolide (TP)/paeoniflorin (Pae) needle tips and BP-hydrogel needle bottoms were generated. TP, as the main component of *T. wilfordii*, has excellent anti-inflammatory, detoxification, and immunomodulatory effects [9,42,43], while Pae is an important extractive of *P. lactiflora*, which can further enhance the therapeutic effect and decrease the toxicity of TP [44–46]. When the traditional Chinese medicine integrated responsive MNs were pasted on the skin, their mechanical properties could bring about instant skin penetration to fully contact the lesion regions. Benefiting from the two-layer structure's dominance of the composition dispersion, their loaded drugs could be quickly released under the NIR by the photothermal regulation capability of the BP components [47–52]. Based on these features, the traditional Chinese medicine integrated responsive MNs have shown excellent performance in the treatment of skin thickening and hardening, collagen fiber increase, and capillary dilatation in the early stages of SSc mouse models. These results indicated that the proposed Chinese medicine integrated responsive MNs had clinical significance in treating early SSc and enormous potential for promoting many other diseases.

## Results

### Morphology characterization of compound MNs

**Fig. 2.**
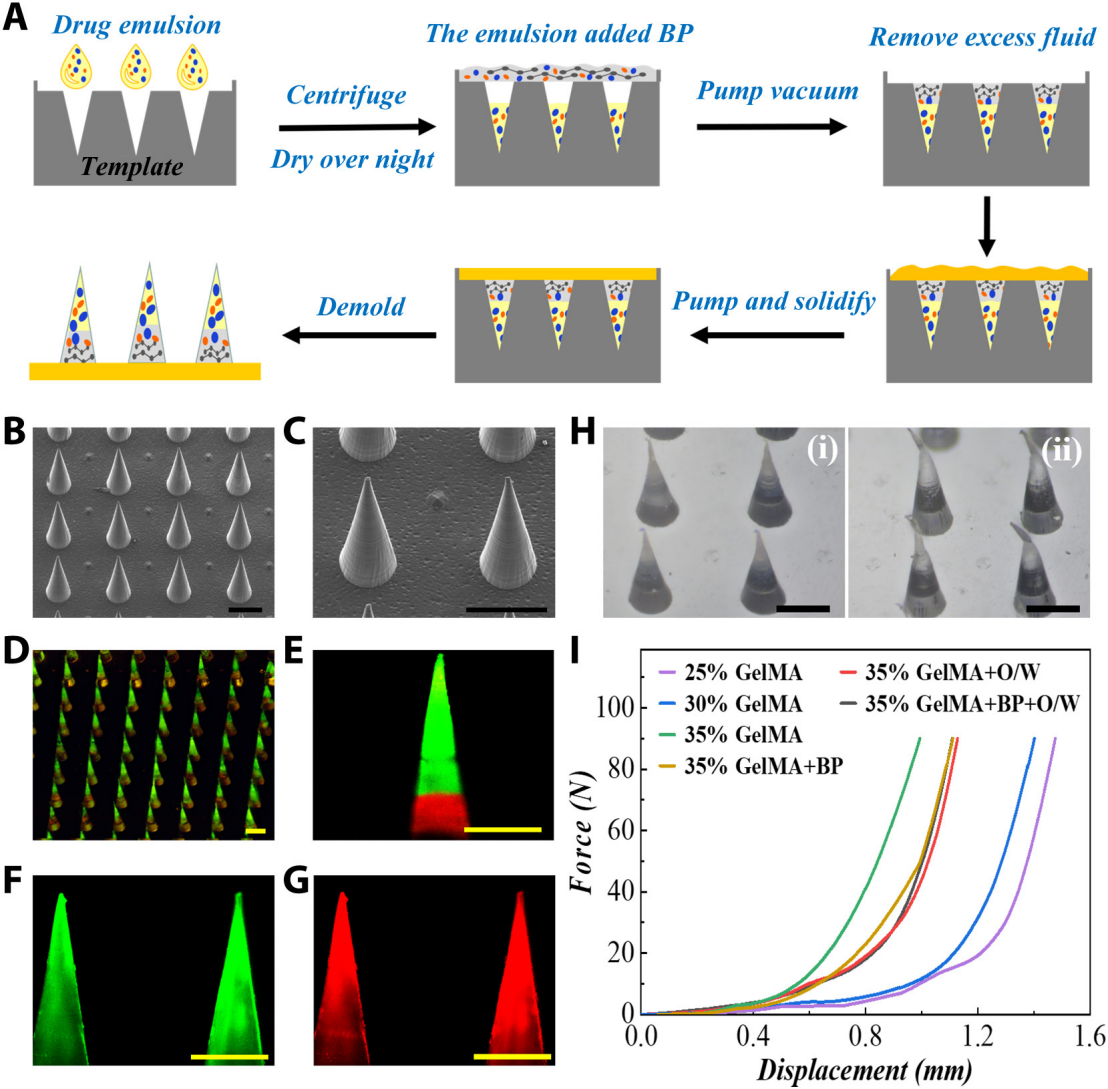
Characterization and mechanical strength of the MNs. (A) Schematic illustration of the fabrication of the photoresponsive MNs. (B and C) SEM images of the MNs. (D) Fluorescence microscopy images of the MN arrays loaded with fluorescent nanoparticles. (E) Confocal images of MNs loaded with fluorescent nanoparticles to characterize the structure of tips. (F and G) Confocal images of MNs loaded with fluorescent nanoparticles to characterize two drugs. (H) Optical images of MNs before being pressed and after. (I) Forces of the MNs with different GelMA concentrations and compositions. Scale bars, 500 μm.

In a typical experiment, we used a layer-by-layer curing method to prepare the MNs in three steps (Fig. 2A). Methacrylate gelatin (GelMA) with good cell activity and biocompatibility was selected as the base material of the tip, and the tip was divided into two layers. The base material only containing drug emulsions was filled into the upper layer of the tip by centrifugation and dried overnight at 37 °C. Then, the base material with BPs was quickly filled into the remaining tip by vacuuming. After the redundant material was removed and the tip was solidified, polyethylene (glycol) diacrylate (PEGDA) as the backing material covered the tips and was cured under ultraviolet (UV) light. Finally, the complete MN patch was obtained by drying overnight at 37 °C and careful demolding. In this way, the functional structure of MNs was arranged in layers. It was measured that the sharp tip of the MNs had a cone shape with a height of 950 μm and a bottom diameter of 430 μm (Fig. S1). The overall view of the MNs was further represented by scanning electron microscopy (SEM) (Fig. 2B and C) and fluorescence microscopy (Fig. 2D). Besides, by adding fluorescent nanoparticles of two colors in the layered fabrication, the double-layer structure of the MN could be observed under a confocal microscope (Fig. 2E). This double-layer design ensured that the maximum of medicine was released into the deepest layer of the skin and that the photothermal conversion could be easily completed at the bottom. In addition, we dispersed differently colored fluorescent nanoparticles homogeneously in water and oil in a separate experiment to simulate the coloading of two drugs and their distributions, and emulsified TP could be distributed equally in MNs (Fig. 2F and G).

The MN tips need to have adequate mechanical strength and puncture ability, which are related to their material compositions. To optimize the material composition, we tested the maximum force that the GelMA MNs with different hydrogel concentrations and compositions could tolerate. When measuring, the MNs were put on the horizontal device of the electronic universal testing machine, with the tip pointing upward and the force sensor element slowly pressed to the MNs. The recording begins when the sensor was exposed at the top and ends when the force reached 90 N. Results showed that the mechanical strength of MNs enhanced as GelMA concentration increased (Fig. 2H and I). The stress–displacement curve showed that a single needle of MNs could sustain a force of more than 0.9 N, which was enough to pierce the skin. Meanwhile, it was found that the mixed material of BP and drug emulsion also affected the mechanical strength of MN tips. Considering the retention time and the pathological skin characteristics of SSc, we selected 35% GelMA with the best mechanical strength. We also made puncture experiments on pig skin, and SEM images showed that MNs could pierce the pig skin successfully (Fig. S2).

### Performance of photothermal conversion

**Fig. 3.**
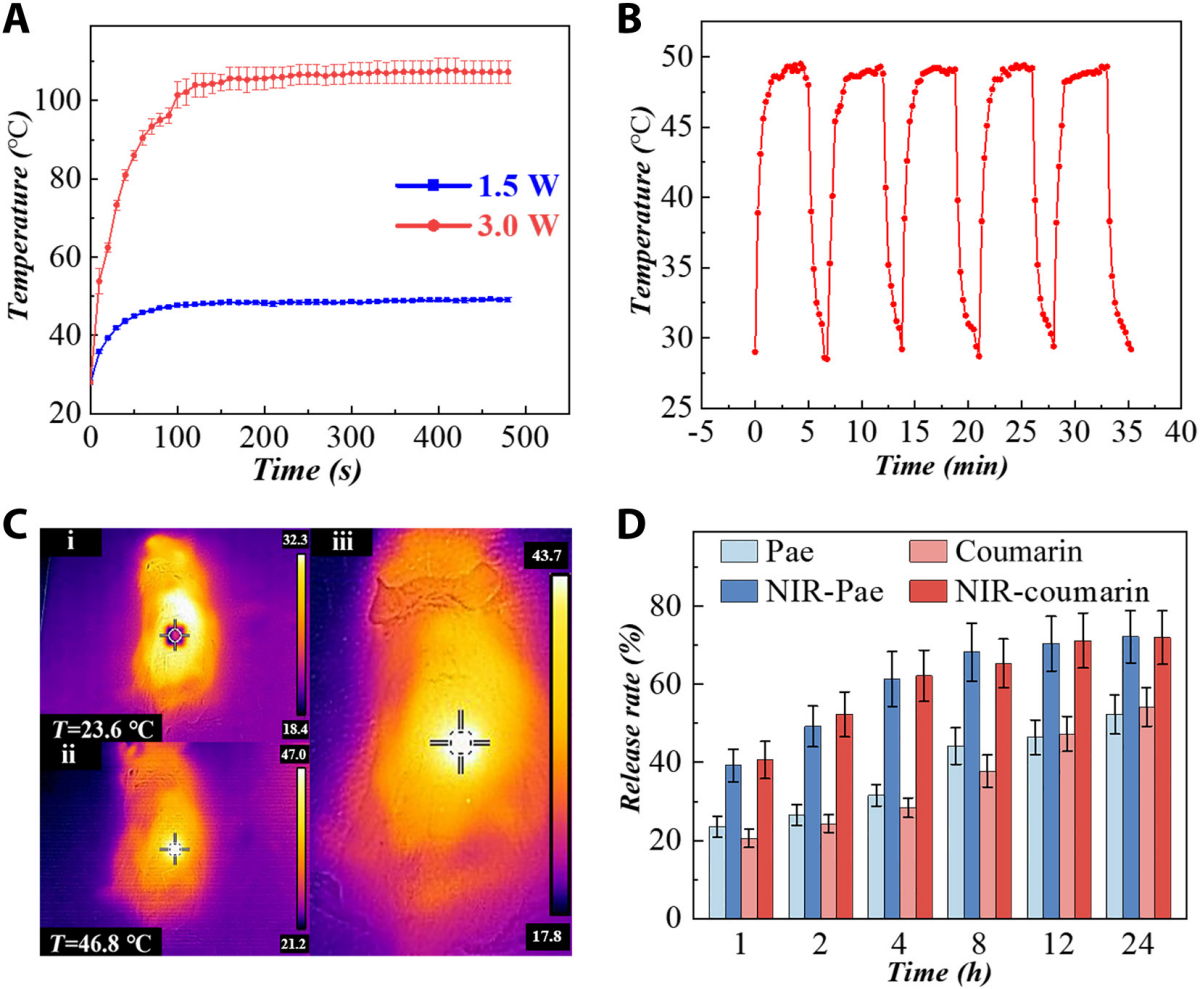
Photothermal conversion and controllable drug release. (A) Temperature rising profiles of the MNs under NIR light of 1.5 and 3.0 W. (B) Temperature changes of MNs during 5 on/off cycles. The power of NIR light was 1.5 W. (C) Thermal images of MNs applied to the mouse dorsal skin before and after 2-min NIR irradiation. The NIR power was 1.5 W. (D) Drug-releasing conditions of different groups (BSA group, NIR-BSA group, coumarin group, and NIR-coumarin group).

The loaded BPs gave the MNs the capacity to emit heat under NIR illumination. We tested the photothermal properties of BPs at different concentrations controlled by high and low power (Fig. S3). It is worth noting that 1.5 W NIR power could rapidly increase MN temperature to a peak of 49 °C within 3 min, as shown in Fig. 3A. While the rate of work was increased to 3 W, the temperature of MNs rapidly reached 100 °C in 2 min. The BP concentrations also influence the photothermal conversion effects, with higher BP concentrations leading to a higher temperature. Taking account of these results and the possible skin injury caused by overheating, 1.5 W and 0.2 mg/ml were perceived as the optimum conditions. To verify that this photothermal conversion capability can be repeatedly triggered by 1.5-W NIR light, we performed five continuous NIR switching cycles on MNs. In the five cycles, the conversion capacity of MNs from heating to cooling did not perform significant attenuation (Fig. 3B). These performances illustrated that the photothermal conversion process of MNs could be repeated under the control by NIR. The MNs not only could generate heat triggered by NIR in vitro but also were applied to the mouse skin. The thermal imager observed a rapid rise in skin temperature from 28 °C to about 47 °C within 2 min, exhibiting desirable photothermal conversion in vivo (Fig. 3C).

### Drug release behavior of MNs

In terms of drug release, the release profiles of two kinds of drugs from the MNs and the impacts of NIR were measured. We applied coumarin with similar molecular weight and physicochemical properties to simulate TP. We observed that the release of both drugs basically achieved 70% of the drug load under 10 min of NIR per hour, while those without NIR simulation only reached 50%. These results confirmed the drug delivery ability and responsive release effects of MNs (Fig. 3D). In addition, to verify the effect of drug emulsification on release, Pae was loaded into the MNs in emulsified and non-emulsified ways, respectively. It was found that the emulsified drug release was higher and faster in comparison to the non-emulsified one (Fig. S4). Especially, after NIR irradiation, the drug release rate was accelerated to a higher degree, and the total drug release increased significantly. We also compared the release of 5 and 10 min of NIR per hour in each group. There was little difference in the total release amount between the two modes, and the release rate was a little faster in the 10-min irradiation group (Fig. S4). These results verified the effective control of BP-loaded responsive MNs on drug release.

### Biocompatibility of MNs and synergies between drugs

**Fig. 4.**
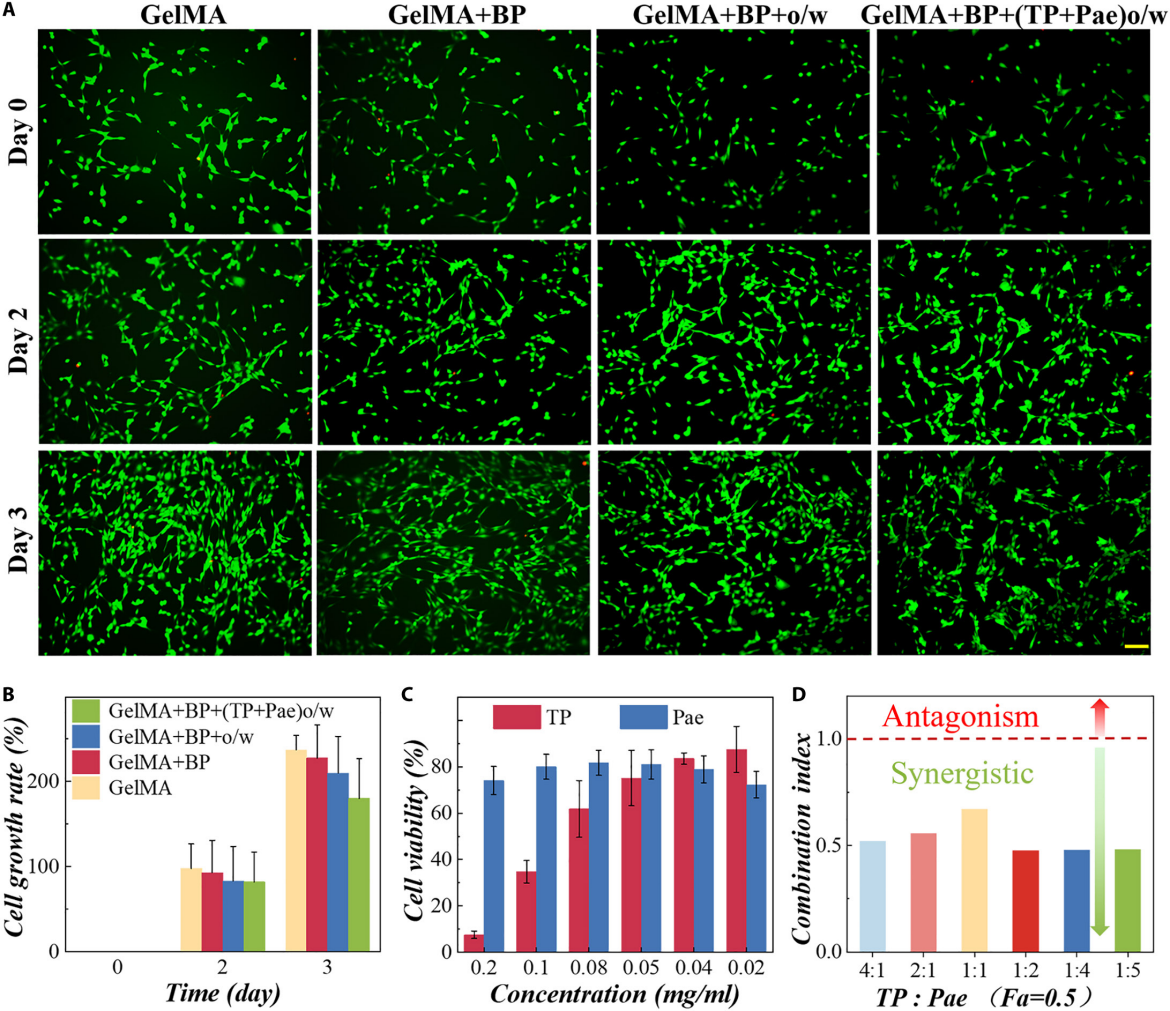
Biocompatibility of the MN patch, toxicity of drugs, and synergy between two drugs. (A) NIH-3T3 cells cocultured with GelMA MNs, GelMA + BP MNs, GelMA + BP + o/w MNs, and GelMA + BP + (TP + Pae)o/w MNs at days 0, 2, and 3. Scale bar, 100 μm. (B) Growth rate of NIH-3T3 cells cocultured with materials for 3 days. (C) CCK-8 assay of NIH-3T3 cocultured with TP and Pae. (D) CI values at Fa = 0.5 of different combinations of TP with Pae (CI value >1 indicates antagonism, and CI value <1 is the synergistic effect).

As for the biocompatibility of MN tips, it was observed that standard fibroblast cell line (3T3) cells grew well cocultured with MN tip composition, demonstrating favorable biosafety (Fig. 4A and B and Fig. S5). We also tested the degradation of GelMA hydrogel in 72 h (Fig. S6) and found that GelMA hydrogel could remain stable in vitro and vivo for several days. To test the cytotoxicity and synergy of the two drugs, we employed the CCK-8 assay to test the viability of 3T3 cells cultured with different concentrations of TP and Pae (Fig. 4C). A similar protocol was used to verify the performance of different water–oil ratios on cell viability (Fig. S7). The proliferation rate of cells reflected that TP concentration was 0.02 mg/ml and 0.02 to 0.1 mg/ml of Pae had little cytotoxicity. Castor oil share also affected the survival rate of cells, and it displays little cytotoxicity when the ratio of water to oil was 20:1. To depict the synergetic effects, combination index (CI) could quantitatively determine the strength and quality of drug interactions was used through the Chou–Talalay method. Specifically, CI < 1 indicates synergistic effect, and the synergistic effect is stronger when CI value is lower. The results are illustrated by Fa-CI (Fa: inhibition rate) (Fig. S8). The CI values at Fa = 0.5 of different combination treatments were summarized in Fig. 4D. A synergism effect was observed for the TP/Pae ratio from 4:1 to 1:5 in 3T3 cells. We also found that the cell growth of the Pae + TP group was better than that of TP alone when the concentration of TP was 0.02 mg/ml and TP:Pae was 1:2 (Fig. S8), which could confirm the detoxifying effects of Pae on TP and the advantages of combinative administration.

### Efficacy validation on SSc mouse models

**Fig. 5.**
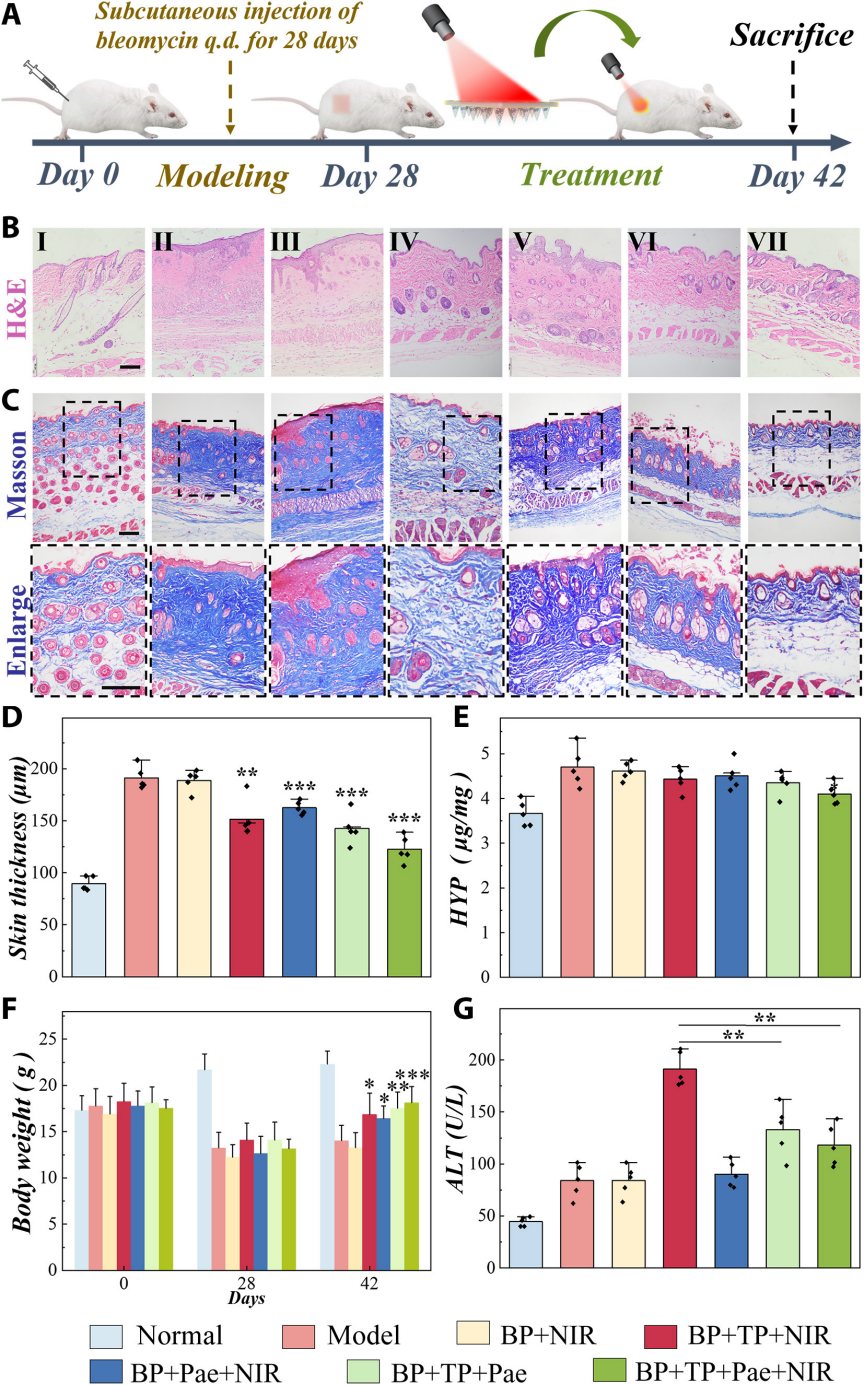
Model establishment and therapeutic effect/biotoxicity of the responsive MNs on SSc model mice. (A) Process of the establishment and treatment of the SSc mouse model. (B) Corresponding H&E staining of the skin lesions of different groups on day 42. (C) Collagen deposition illustrated by Masson’s trichrome staining of different groups on day 42. (D) Skin thickness of the mice in corresponding groups. All statistical differences were compared with the model group. (E) Concentration of HYP in different groups on day 42. Statistical differences were compared with the model group. (F) Body weight of the corresponding groups at different stages during the induction and therapy of bleomycin-induced SSc model mice. Statistical differences were compared with the corresponding groups on day 28. (G) Alanine transaminase (ALT) levels in the blood of mice. (I: normal group; II: model group; III: BP + NIR group; IV: BP + TP + NIR group; V: BP + Pae + NIR group; VI: BP + TP + Pae group; VII: BP + TP + Pae + NIR group). Scale bars, 20 μm. *P < 0.05, **P < 0.01.

To prove the practical value of MNs, we established an early full-thickness skin wound model of SSc mice by daily bleomycin injection and by creating a 1 cm^2^ square wound on their backs, with one group left as the blank control (normal group). Then, the bleomycin-induced model mice were randomized into six groups averagely (Fig. 5A). One group was regarded as the negative control (model group) and received no treatment. In addition, MNs loaded with different combinations of BP, TP, and Pae as well as NIR were applied to other mice as treatment groups, which were the BP + NIR group, BP + TP + NIR group, BP + Pae + NIR group, BP + TP + Pae group, and BP + TP + Pae + NIR group, respectively. Changes in the area of skin injury were recorded and analyzed during the treatment. It was observed that the BP + TP + Pae and BP + TP + Pae + NIR groups, which received TP and Pae at the same time, recovered better than did other groups (Fig. S9). Hematoxylin and eosin (H&E) staining showed that compared with the negative control group, other treatment groups had varying degrees of dermis thickness reduction (Fig. 5B). Among them, the BP + TP + Pae + NIR group displayed the best dermis thickness reduction (Fig. 5D). Besides, collagen synthesis, deposition, and orientation play an important role in the development of SSc. Masson staining and hydroxyproline (HYP) levels showed that the BP + TP + Pae + NIR group reduced the collagen thickness of the lesion skin and the degree of skin fibrosis (Fig. 5C and E and Fig. S10). As an amino acid specific to collagen, HYP levels correlate with the severity of fibrotic lesions. The proline content showed that the BP + TP + Pae + NIR group could decrease the HYP content of the skin lesions and reduce the degree of skin fibrosis. The body weight of mice in treatment groups also increased (Fig. 5F). We also observed the toxicity of the MNs and the attenuated effect about the combination of the two drugs, demonstrating that the MN-loaded TP and Pae simultaneously effectively reduced liver toxicity of TP (Fig. 5G and Fig. S11). At the same time, there was no obvious toxicity to kidney and ovary (Fig. S12).

**Fig. 6.**
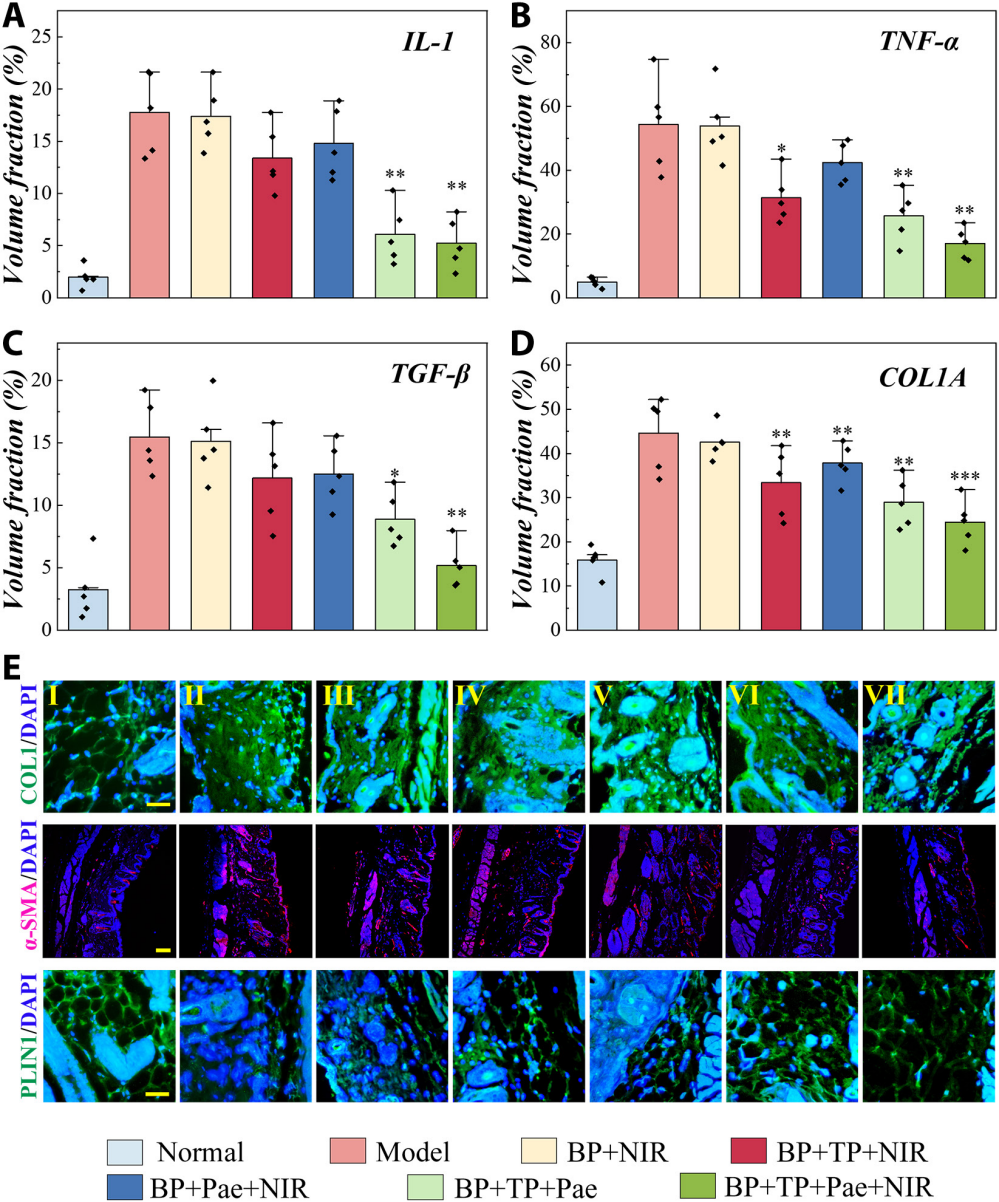
Investigation of the biological mechanism of the SSc mice. (A to D) Fluorescence volume fraction statistics of IL-1 (A), TNF-α (B), TGF-β (C), and COL1A (D). (E) Expression of COL1A, α-SMA, and PLIN1 was detected by immunofluorescent staining in different groups (I: normal group; II: model group; III: BP + NIR group; IV: BP + TP + NIR group; V: BP + Pae + NIR group; VI: BP + TP + Pae group; VII: BP + TP + Pae + NIR group). Scale bars, 20 μm.

Inflammation and angiogenesis were further evaluated to explore the processes and biological mechanism of lesion repair. For this purpose, interleukin-1β (IL-1β) (IL-1), tumor necrosis factor-α (TNF-α), transforming growth factor-β1 (TGF-β1) (TGF-β), COL1A1 (COL1), α-smooth muscle actin (α-SMA), Perilipin-1 (PLIN1), and IL-6 immunofluorescence staining were performed. Only low levels of IL-1 and IL-6 were secreted, and the expression of TNF-α and TGF-β was observed to decrease in treatment groups (Fig. 6A to D and Fig. S13), indicating little sign of inflammation. Besides, immunofluorescence staining of COL1 and α-SMA separately revealed the decreased collagen expression and reduced α-SMA expression in the collagen layer, and we also found that the expression of PLIN1 increased may denote a generation of adipose layer in the treatment groups (Fig. 6E). Additionally, since the BP + TP + Pae + NIR group showed the best performance among all other groups, it could be deduced that the NIR-guided combined delivery of the two drugs had the best therapeutic effect. Notably, we also verified the influence of other parameters that had no relationship with drugs on the disease model and found that NIR or merely MN patch had almost no therapeutic effects on SSc. All these results suggest that responsive MNs have an ideal ability to treat early SSc skin lesions.

## Discussion

In conclusion, we presented photothermal responsive MNs loaded with TP and Pae, which could achieve controlled drug release and treat early SSc skin injury effectively. Due to the high mobility and unclear pathogenesis of SSc, the treatment of SSc is an urgent problem to be solved all over the world. At present, the clinical application of drugs is limited to great adverse effects and restricted potency, bringing great pain to patients. Therefore, exploration of new therapies to control SSc timely in an early stage and to alleviate the suffering of patients is urgently needed. To treat early skin lesions in SSc with minimal side effects, we fabricated TP/Pae-loaded responsive MNs for SSc skin lesion treatment. GelMA, which was biocompatible with excellent mechanical strength, was chosen as the MN tip material with TP/Pae encapsulation in the upper half and TP/Pae/BP in the bottom one. TP played an important role in immunosuppression and skin injury, while Pae had excellent immunomodulatory ability and excellent performance in reducing the side effects of TP. The combination of TP and Pae was particularly effective in regulating immunity and improving skin fibrosis. Due to the good photothermal effect of BPs, the local temperature of the affected skin increased rapidly under NIR irradiation, accelerating the release of emulsified drugs. It was worth noting that our MNs presented good utility performance in the treatment of early full-thickness skin wound model of SSc mice, indicating that they would have broad prospects in early scleroderma and related biomedical areas.

## Materials and Methods

### Materials

PEGDA, anhydride (94%), and gelatin (from porcine skin) were obtained from Sigma-Aldrich. Gelatin methacryloyl (GelMA) was synthesized by methacrylic anhydride and gelatin in the laboratory. Castor oil, Tween 20, hydroxy-2-methylpropiophenone (HMPP), TP, and Pae were purchased from Aladdin Industrial Corporation. BP was purchased from Nanjing XFNANO Material Tech Co. Ltd. Fetal bovine serum (FBS) and penicillin–streptomycin double antibiotics were provided by Gibco. Cell Counting Kit-8 (CCK-8) and Dulbecco’s modified Eagle’s medium (DMEM) were purchased from KeyGEN BioTECH Ltd., Jiangsu. Calcein AM cell viability assay kit/propidium iodide (PI) was obtained from Beyotime Biotechnology. The antibodies including α-SMA, IL-1, IL-6, TNF-α, and TGF-β were purchased from Servicebio. The antibodies PLIN1 and COL1 were obtained from Abcam. Phosphate-buffered saline (PBS) was prepared in the laboratory. All reagents and drugs were of standard-based analytical levels and immediately used as received.

### Characterization

We used a stereomicroscope (JSZ6S, Jiangnan Novel Optics) to observe the morphology of MNs under the bright field and captured by a charge-coupled device camera (Oplenic digital camera). SEM images were characterized by a scanning electron microscope (HITACHI SU8010). The fluorescence photographs were observed by a fluorescence microscope (Olympus, IX73-A12FL/PH) with the pictures taken by charge-coupled device camera. The fluorescence images of the MNs were obtained with a laser scanning confocal microscope (Nikon, A1). The mechanical properties of MNs were tested with All-purpose Electronic Tester (Instron 5944). The thermograms were recorded by thermography (FLIR, E5xt).

### Cell lines and animals

The cells used in experiments were standard fibroblast cell line (NIH-3T3) cells provided by the Cell Repository of the Chinese Academy of Sciences and incubated in DMEM added with 1% (v/v) penicillin–streptomycin double antibiotics and 10% (v/v) FBS in a cell incubator with the conditions of 37 °C, 5% CO_2_. Female BALB/c mice (20 to 25 g) were obtained from SPF (Beijing) Biotechnology Co. Ltd. All experiments related to the handling and caring of animals were finished with the *Guide for the Care and Use of Laboratory Animals* in strict accordance and conformed with ethical review from Animal Investigation Ethics Committee of Nanjing Drum Tower Hospital.

### Fabrication of the MNs

To prepare a 2 mg/ml stock solution, 4 mg of TP was dissolved in 2 ml of methanol. GelMA (0.35 g/ml), BP (0.2 mg/ml), TP (0.2 mg/ml), Pae (0.2 mg/ml), and HMPP (1%, v/v) were mixed with castor oil and emulsified with Tween 20 at 20:1. In the same way, another solution without BPs was prepared as the MN tip material. Tip solution (100 μl) without BP was added into the MN template with a pipetting tube, which filled into the cavity of the template by means of horizontal centrifugation. Extra tip solution was removed with about one-half of the tip being filled, and it was dried in oven for 37 °C overnight. Tip solution with BP was then added, which quickly filled the remaining cavity with vacuum and was cured afterward with 10-W UV irradiation for 20 s. The tips were covered with 150 μl of back layer solution completely, and the tip was irradiated by UV for 12 s and then dried in drying oven (37 °C) for 12 h. Finally, the mold was carefully detached to obtain the complete MNs.

### Mechanical strength tests

The MNs with different concentrations of GelMA and other components were placed on a horizontal position of the electronic universal testing machine (Instron 5944) in turn with the tips toward the pressure sensor. Subsequently, the pressure transducer vertically pressed down the MNs at the rate of 2 mm/min. The measurement of force started from the pressure transducer just touching the MN and finished with the compression force reaching the measuring range of the testing machine.

### NIR-triggered photothermal conversion capability

In the photothermal conversion experiment, the MNs were put under an 808-nm light at a distance of 5 cm with a power of 1.5 or 3.0 W. Real-time temperature of the MNs was collected every 10 s. For the on/off cycles, NIR irradiation was applied until the MNs reached the maximum temperature, and then the next cycle was begun when it cooled to the lowest point (room temperature). The initial and final thermal images of the MNs applied to mice skin were also taken.

### Biocompatibility experiments

Alcohol (75%) is used to disinfect the MN patches. These patches were rinsed 3 times with PBS and then sterilized with ozone and UV rays overnight. NIH-3T3 cell suspension at a concentration of 2 × 10^4^ cells/ml was evenly divided into 4 groups. Each group of 500-μl cell suspension was coincubated with different kinds of stuff and drugs in the 48-well plate (Corning, USA) for 72 h. Group 1 was 35% GelMA films; group 2 was GelMA + BP films; group 3 was GelMA + BP + o/w films; group 4 was GelMA + BP + (TP + Pae)o/w films. All the films were in the same size, and each group had four parallels. Cell proliferation was observed with an inverted fluorescence microscope after staining with calcein AM/PI on days 0, 2, and 3.

### Drug toxicity tests

Cell suspensions (200 μl) at a concentration of 2 × 10^4^ cells/ml were cocultured with TP and Pae of different concentrations (0.02, 0.04, 0.05, 0.08, 0.1, and 0.2 mg/ml) separately in 96-well plates for 12 h, and each group had six parallels. The original medium was then changed to new medium mixing 10% CCK reagent, and the cells were incubated for 2.5 h for color development. Then, the absorbance value was measured at 450 nm.

### Analysis of TP-Pae interactions and efficacies

TP and Pae were mixed in different ratios of 4:1, 2:1, 1:1, 1:2, 1:4, and 1:5; at the same time, six concentration gradients were set between 0.02 and 0.2 mg/ml of TP. They were cocultured with NIH-3T3 cells for 12 h, and then cell viability was evaluated by CCK-8 assay (four parallels for each group). The Chou–Talalay (Compusync software) CI was used to calculate and detect whether the interaction between TP and Pae was synergistic, additive, or antagonistic. The character and intensity of drug interactions can be quantitatively determined by CI value (CI > 1 is antagonistic, CI = 1 indicates additive, 0.7 < CI < 1 marks a slight synergistic effect, 0.3 < CI < 0.7 indicates synergy, CI < 0.3 signifies a strong synergy).

### Drug release tests in vitro

To verify the drug release ability, coumarin with similar molecular weight and physicochemical properties was used to replace TP in experiments. MNs loaded with drugs were immersed in PBS, and the release of drugs in PBS was detected at specific time intervals. Six different groups were designed [GelMA + BP + o/w group, GelMA + BP + o/w + NIR group, GelMA + BP + Pae(o/w) group, GelMA + BP + Pae(o/w) + NIR group, GelMA + BP + Coumarin(o/w) group, and GelMA + BP + Coumarin(o/w) + NIR group]. The NIR irradiation group was irradiated for 10 min every half hour. Four parallel tests were conducted for each group, and the drug release was recorded at 0, 1, 2, 4, 8, 12, and 24 h, respectively.

### Establishment of mouse model and treatment

Forty-nine female mice (BALB/c) were randomly and equivalently divided into seven groups and injected with 0.1 ml of bleomycin (Nippon Kayaku Co. Ltd., Japan) at a concentration of 1 mg/ml in normal saline every day for 4 weeks to induce skin fibrosis [53]. Then, on the 29th day, one mouse was randomly selected from each group and killed to observe the modeling situation. The seven groups were normal mice without modeling (normal group), disease mice without treatment after modeling (model group), BP + o/w + NIR MN patch treatment (BP + NIR group), BP + TP(o/w) + NIR MN patch treatment (BP + TP + NIR group), BP + Pae(o/w) MN patch treatment (BP + Pae + NIR group), BP + TP + Pae(o/w) MN patch treatment (BP + TP + Pae group), and BP + TP + Pae(o/w) + NIR MN patch treatment (BP + TP + Pae + NIR group). All the NIR groups were irradiated for 5 min four times a day; then, the MN patches were removed after attachment for 12 h. After 14 days for treatment, the mice were sacrificed after eyeball blood collection. The skin and organs were collected and soaked in 4% paraformaldehyde. The tissue samples were dehydrated using 70% to 100% graded ethanol, vitrified by dimethylbenzene, embedded by mineral wax paraffin, and sectioned for further histological chemical analysis and immunofluorescence staining.

### Toxicity behavior of the MNs in vivo

We weighed the mice in each group before modeling (day 0), after modeling (day 28), and after treatment (day 42) and recorded the changes in body weight. After the mice were sacrificed, liver, kidney, and ovary were carefully separated to weigh each organ. Then, the main organs were embedded in wax paraffin and carved into 5-μm slices for H&E staining. The blood level of alanine aminotransferase (ALT) of mice in each group was detected with an ALT activity detection kit (Solarbio Science & Technology Co. Ltd., Beijing, China).

### HYP, H&E, Masson staining, and immunofluorescence

Skin tissue (0.05 g) was used to detect HYP content with a HYP content detection kit (Solarbio Science & Technology Co. Ltd., Beijing, China). Tissue sections (5 μm) were prepared for H&E and Masson staining, and the epidermal thickness and collagen thickness were statistically analyzed by ImageJ software. Skin tissue (7 μm) was cut for immunofluorescence staining (α-SMA, COL1, PLIN1, IL-1, IL-6, TNF-α, TGF-β) and then analyzed statistically with ImageJ software.

### Statistical analysis

Statistical analysis was deduced by GraphPad Prism software. All data were expressed as average ± SD. Statistical significance was calculated by using Student’s *t* test for comparison between groups.

## Data Availability

The data are available from the authors upon a reasonable request.
