# Supplementary material for: Traditional Chinese Medicine Integrated Responsive Microneedles for Systemic Sclerosis Treatment
Source: Research (Wash D C). 2023 May 9;6:0141. doi: 10.34133/research.0141 (PMC10204745; doi:10.34133/research.0141)
Supplement: Supplementary Materials — Figs. S1 to S13 [file research.0141.f1.docx]

Supporting Information

**Traditional Chinese medicine integrated responsive microneedles for systemic sclerosis treatment**

Xi Luan ^1^, Xiaoxuan Zhang ^2^, Min Nie ^1^ and Yuanjin Zhao ^1, 2,^ *

^1^Department of Rheumatology and Immunology, Nanjing Drum Tower Hospital, School of Pharmacy, Clinical College of Traditional Chinese and Western Medicine, Nanjing University of Chinese Medicine, Nanjing, 210023, China

^2^State Key Laboratory of Bioelectronics, School of Biological Science and Medical Engineering, Southeast University, Nanjing 210096, China

*Corresponding author. E-mail: yjzhao@njglyy.com


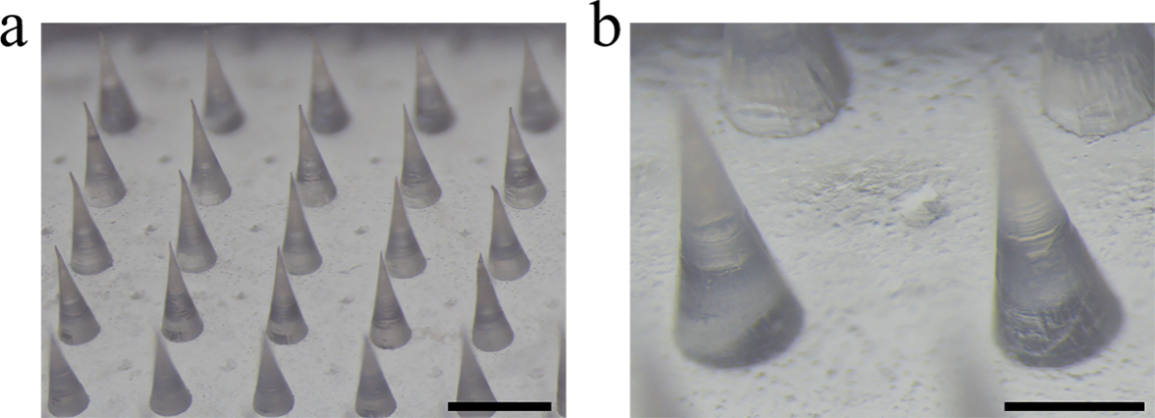


**Fig. S1.** Morphology characterization of MNs. **(a)** Micrograph of an MN array; **(b)** micrograph of double-layer MNs. Both the scale bars are 500 μm.


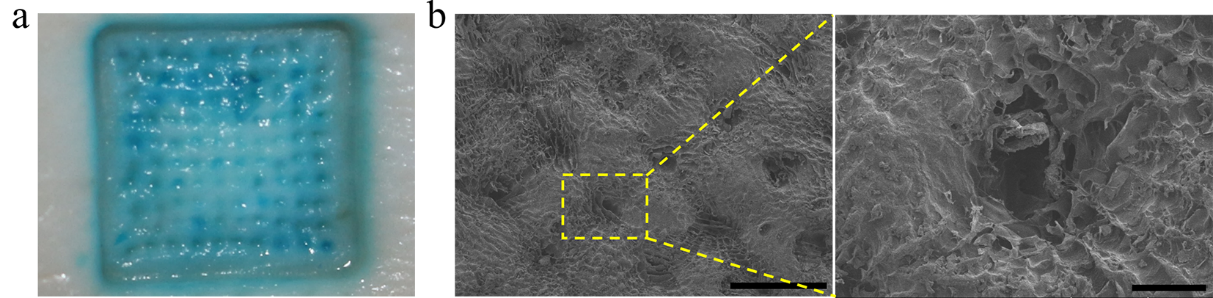


**Fig. S2.** Puncture experiment of MNs on pigskin. **(a)** Picture of defrosted pig skin after a microneedle puncture; **(b)** SEM photographs of the hole remained on the Pig skin after MNs punctured. The scale bars are 1mm and 400 μm, respectively.


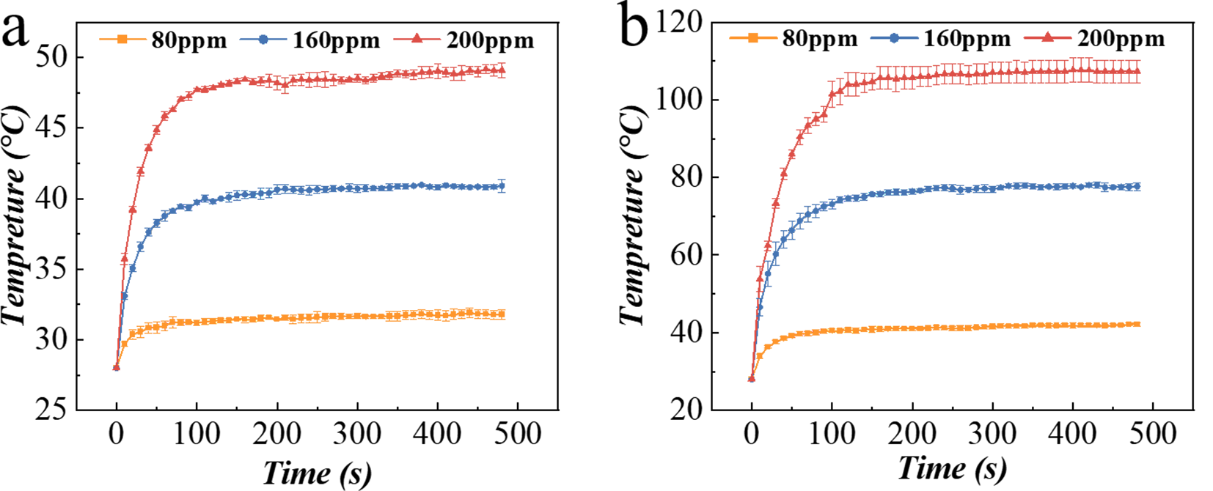


**Fig. S3.** Temperature rising profiles of the MNs loaded with different BP concentrations under NIR light of 1.5 and 3.0 W. **(a)** P=1.5W, **(b)** P=3.0W.


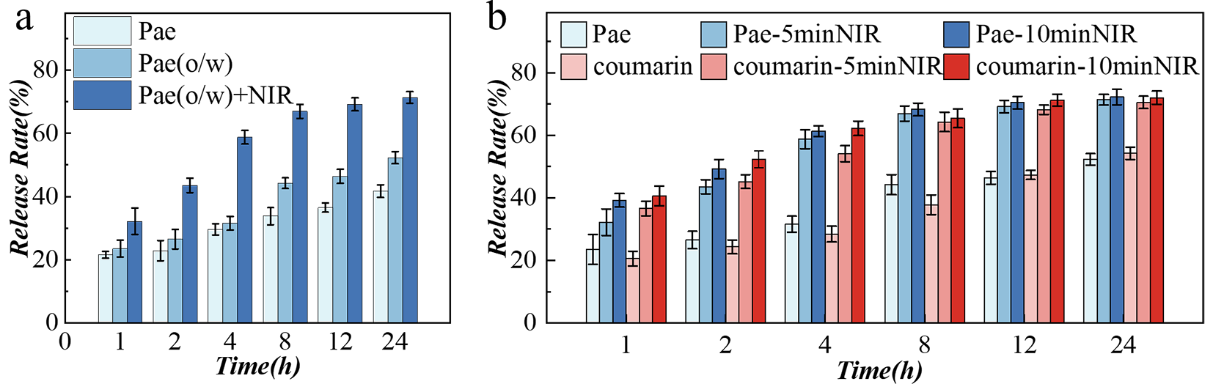


**Fig. S4.** The release rate of drugs in different states. **(a)** Release of drugs loaded into the microneedles in emulsified and non-emulsified ways; **(b)** Release of 0 minutes, 5 minutes and 10 minutes of NIR exposure per hour in each group.

**
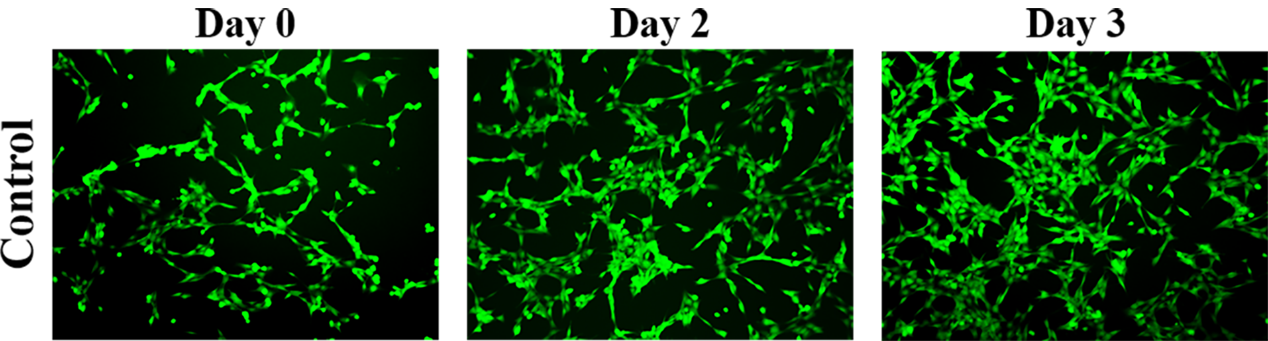
**

**Fig. S5.** NIH-3T3 cells grew in the control group on day 0, day 2 and day 3.

**
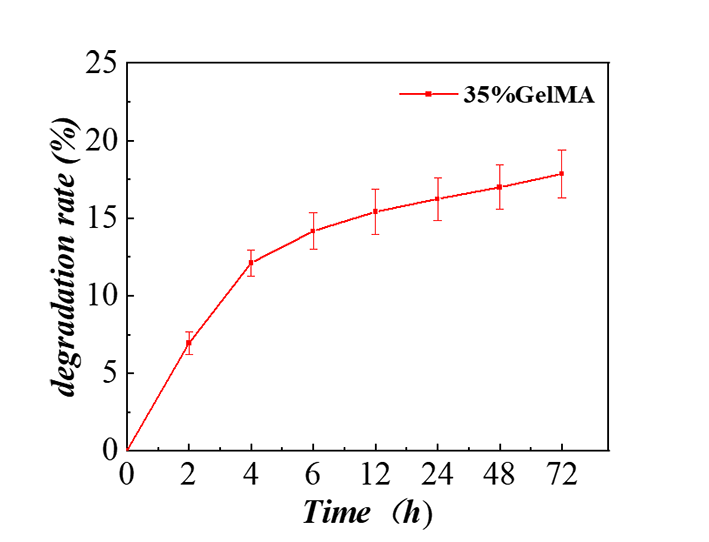
**

**Fig. S6.** The degradation rate of 35% GelMA in 72h.

**Fig. S7.** NIH-3T3 cells viability on different water-oil ratios.


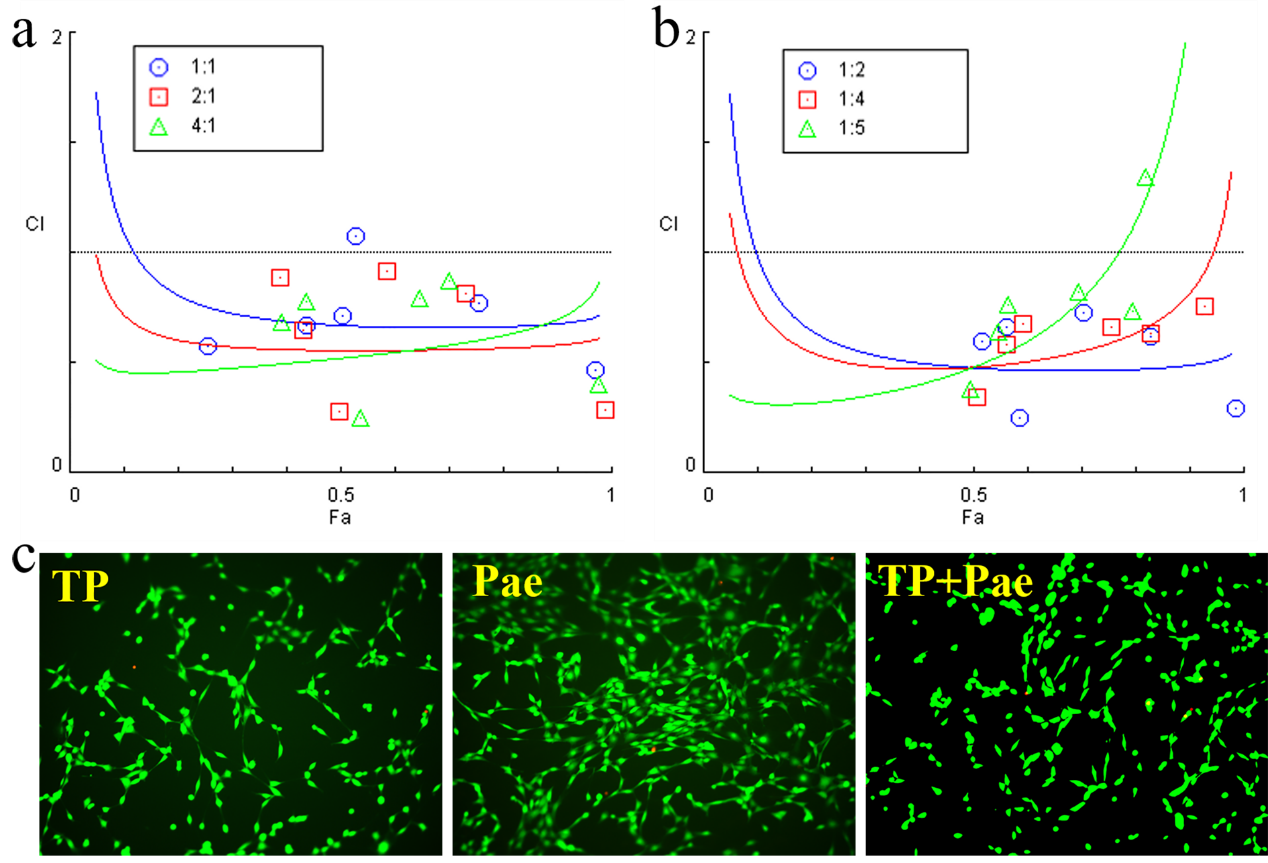


**Fig. S8.** The synergistic effect between two drugs. **(a)** Fa-CI diagram when TP: Pae was on the ratio of 1:1, 2:1 and 4:1; **(b)** Fa-CI diagram when TP: Pae was on the ratio of 1:2, 1:4 and 1:5; **(c)** NIH-3T3 cells co-cultured with TP, Pae, TP+Pae after 12 hours.

Chou-Talalay is a frequently used approach for analyzing medication synergy quantitatively. In theory, drug interactions in vivo will result in constant changes in the pharmacokinetic and pharmacodynamic parameters of the medications, which will represent the drug interactions qualitatively and quantitatively. Based on this premise, the technique uses the Median-effect equation in the law of conservation of mass as a mediator to build a link between medications and the main and multistage kinetics of drug metabolism. Finally, the given combination index (CI) can be used not only for quantitative descriptions of the additive effect, synergism, and antagonism of the combined medicine but also for qualitative descriptions of the effect at the individual level.

In this study, we compare the dose-effect relationship data acquired by cck8 to describe the qualitative and quantitative impacts of medication combinations. The Dm, m, and CI values were determined by the "CompuSyn" software using Chou-Talalay's mathematical model. The magnitude of CI values can be used to quantify the strength and nature of drug-drug interactions (CI>1 for antagonism, CI=1 for summation, 0.7<CI<1 for moderate synergy, 0.3<CI<0.7 for synergy, and CI<0.3 for strong synergy), as shown in Fig. 4d, Fig. S8.


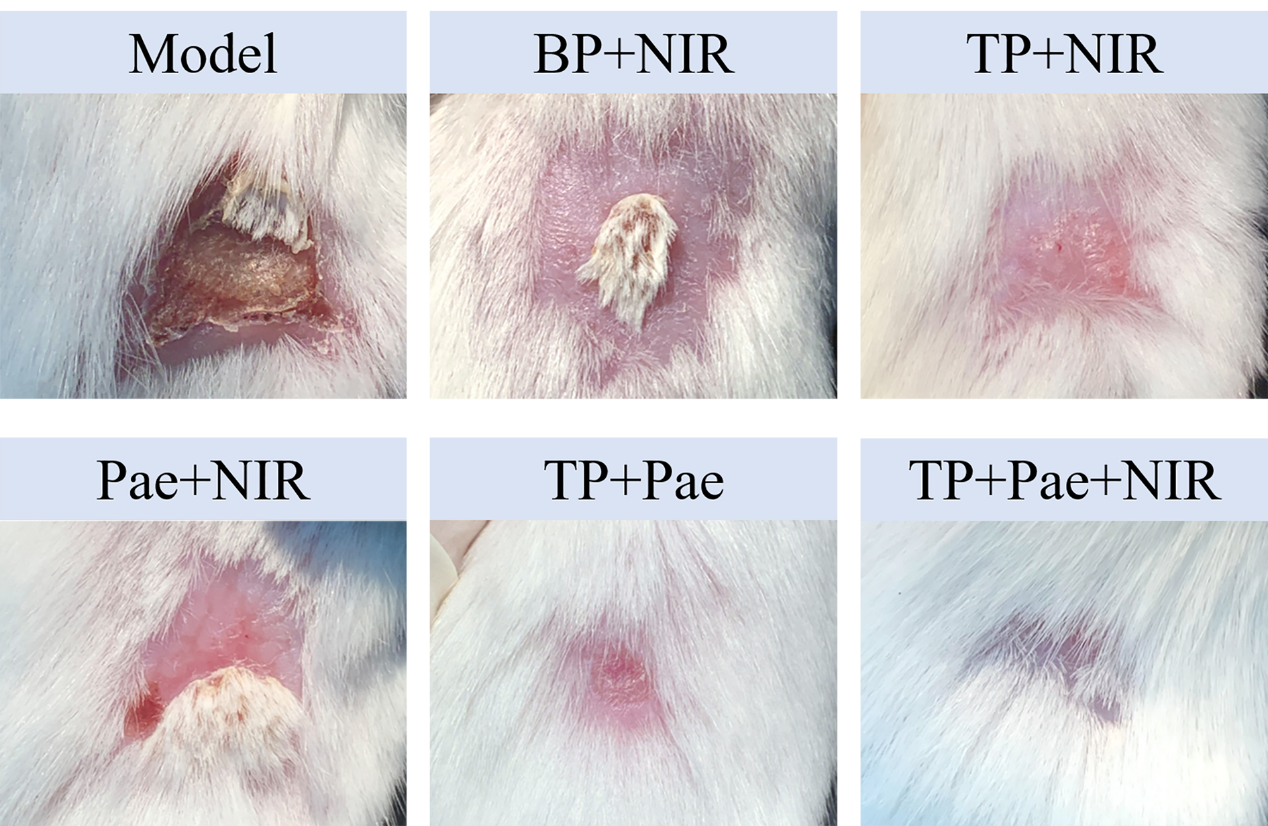


**Fig. S9.** Lesion skin of mice in each group on day 42.

**Fig. S10.** Collagen thickness of mice in each group on day 42. All statistical differences were compared with the model group.


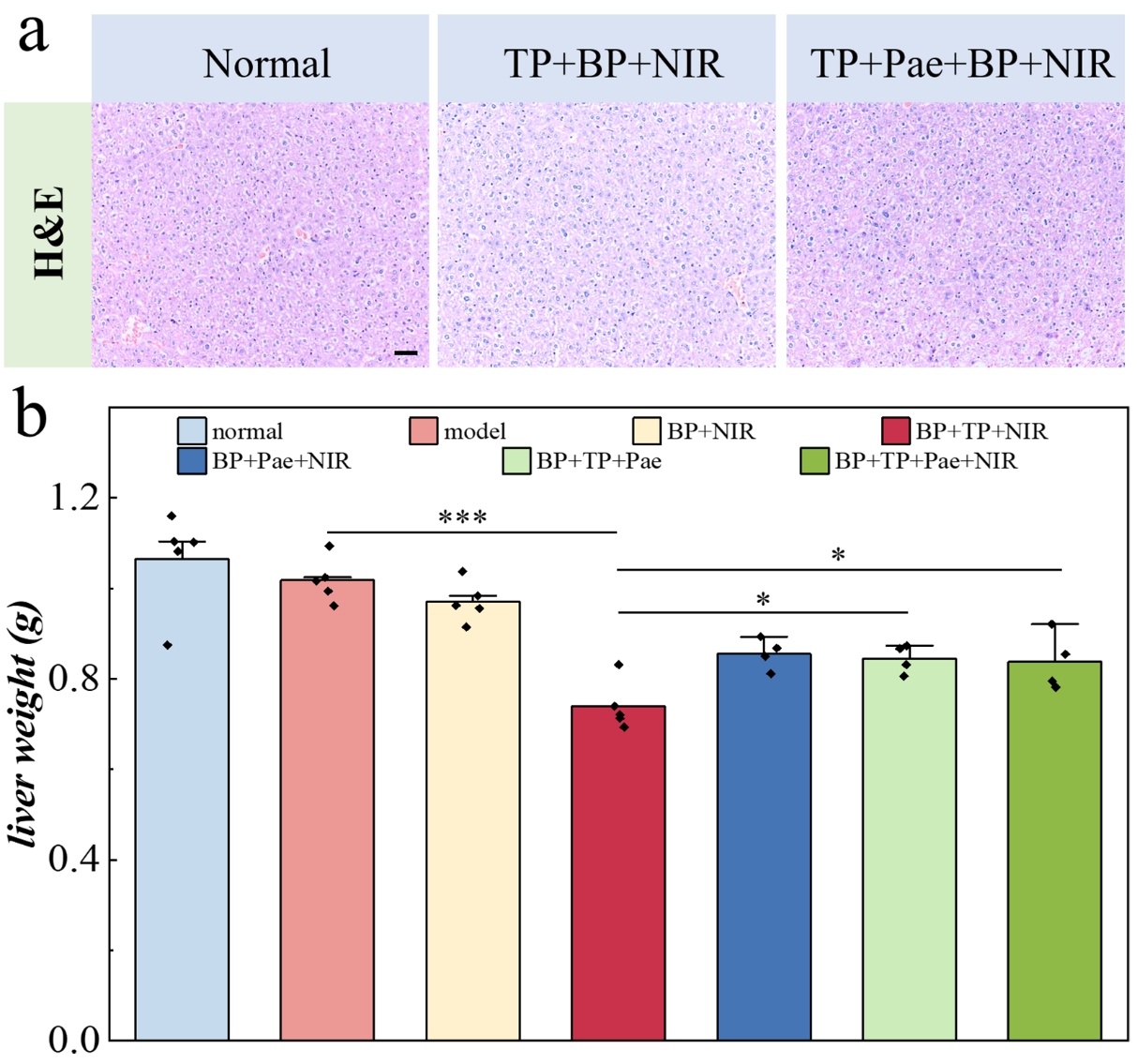


**Fig. S11.** Hepatotoxicity of compound Chinese medicine MNs. **(a)** H&E staining of liver sections. The scale bar is 50 μm; **(b)** Liver wet weights of mice in the corresponding groups after treatment.


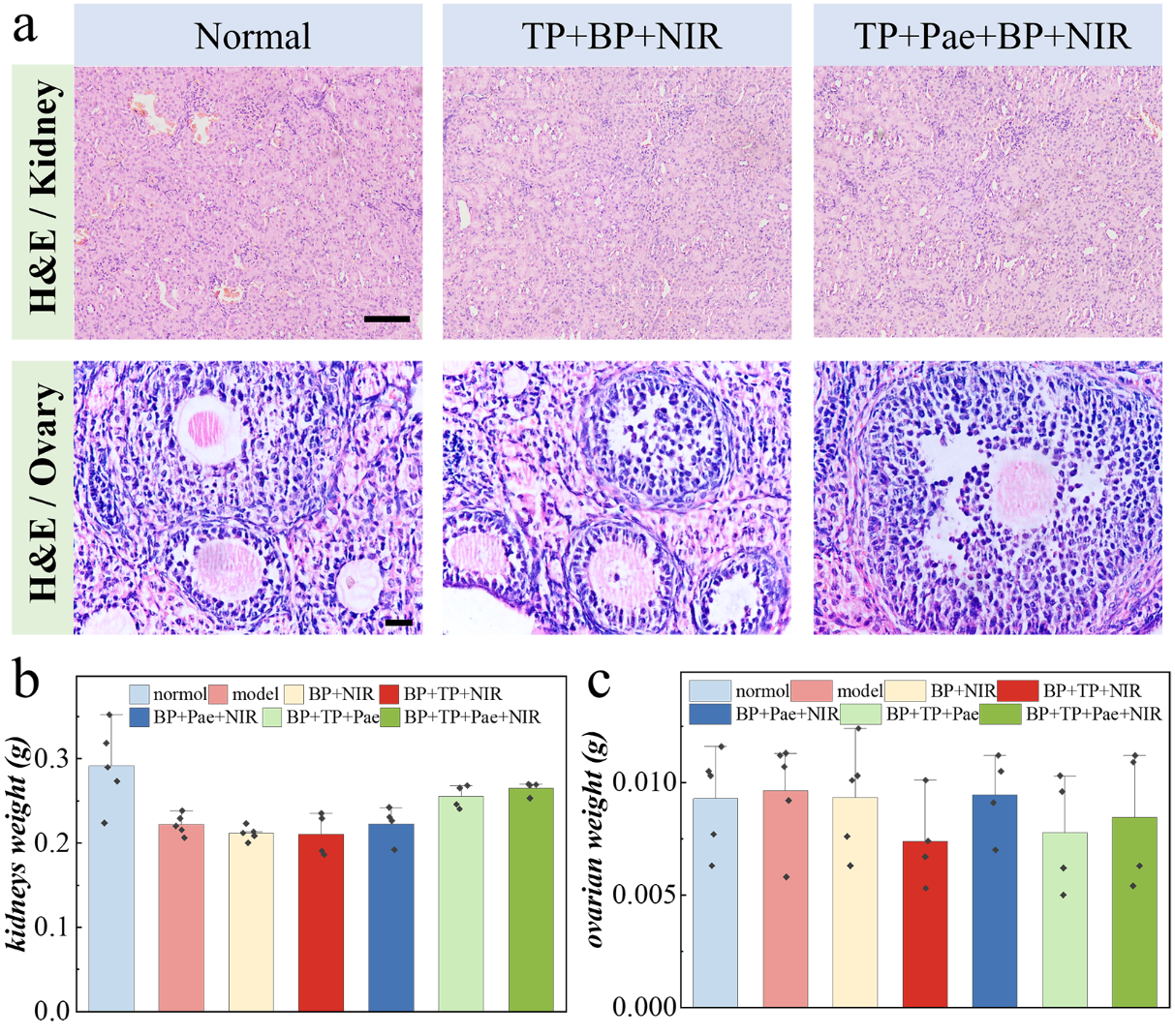


**Fig. S12.** Nephrotoxicity and reproduction toxicity of compound Chinese medicine MNs. **(a)** H&E staining of kidney and ovary sections. The scale bars are 50μm; **(b, c)** Kidney and ovarian wet weights of mice in the different groups after treatment.


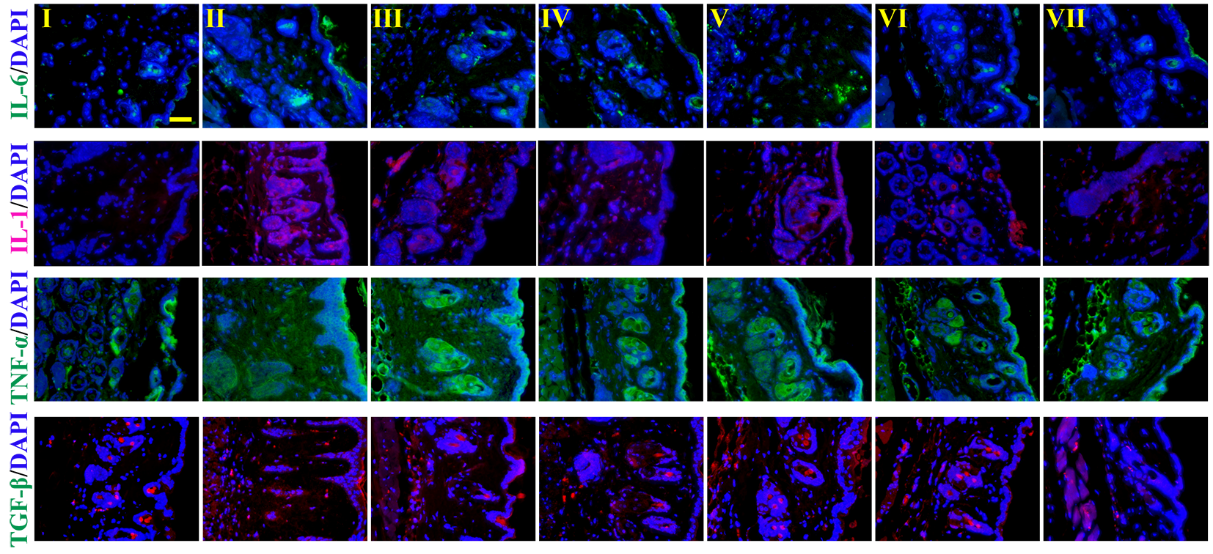


**Fig. S13.** The expression of IL-6, IL-1, TNF-α and TGF-β was detected by immunofluorescent staining in different groups (normal group, model group, BP group, BP +TP group, BP + TP + NIR group, BP +Pae group, BP + Pae + NIR group, BP +TP+Pae group, BP + TP + Pae+NIR group)**.** The scale bar is 50μm.
